# Supplementary material for: Electrochemical direct air capture of CO2 using neutral red as reversible redox-active material
Source: Nat Commun. 2023 Jan 19;14:313. doi: 10.1038/s41467-023-35866-w (PMC9852473; doi:10.1038/s41467-023-35866-w)
Supplement: Supplementary file 1 — Supplementary Information [file 41467_2023_35866_MOESM1_ESM.pdf]

Supplementary Information

Electrochemical direct air capture of CO_2 using neutral red as reversible redox active material

Hyowon Seo and T. Alan Hatton

Contents

1. Supplementary methods

1.1. General information S3

1.2. General material information for electrochemical reaction setups in batch S3

1.3. Procedure for control experiments S4

2. Supplementary Notes

2.1. Setup of NRH/NRH2 redox system for the electrochemical CO_2 capture and release S5

2.2. Setup of NR/NRH2 redox system for the electrochemical CO_2 capture and release in continuous flow S5

3. Supplementary Discussion

3.1. Estimation of the hydroxide anion equivalent S7

3.2. Energy calculation S7

3.3. Evaluation of other dye molecules as redox-active compounds S8

4. Supplementary Figures S10

5. Supplementary Table

5.1. Summary of electrochemical carbon capture methods for direct air capture S24

6. Supplementary References S25

1. Supplementary Methods
1.1. General Information

Material

Commercially available chemicals were purchased from Sigma-Aldrich Chemical Company (Milwaukee, WI), Alfa Aesar (Ward Hill, MA), Acros Organics (Pittsburgh, PA), or TCI America (Portland, OR). All commercially available compounds were used as received. Ag/AgCl reference electrodes were purchased through BASi (West Lafayette, IN). CO_2 cylinders (1, 4, 15, and 100% balanced by nitrogen) were purchased from Airgas (Radnor Township, PA). Purified water was obtained using Milli-Q Direct Water Purification System.

Instrumentation

Proton nuclear magnetic resonance (^{1}H NMR) spectra and carbon nuclear magnetic resonance (^{13}C NMR) spectra were obtained on a Bruker 400 MHz NMR instrument (400 and 101 MHz, respectively). Chemical shifts for proton and carbon are reported in parts per million (ppm). The following designations are used to describe multiplicities: s (singlet), d (doublet), t (triplet), q (quartet), m (multiplet), br (broad). The mass flow controller (GFCS-014657) and flow meter (GFMS-015835) was purchased from Aalborg Instruments & Controls, INC (Orangeburg, NY) and was calibrated for CO_2 by the vendor before use. The CO_2 FT-IR sensor (GC-0016 for 0–100%, GC-0006 for 0–20% of CO_2) was purchased from CO2Meter (Ormond Beach, FL) and was calibrated for CO_2 before use. All cyclic voltammetry (CV) experiments were performed using PARSTAT MC (PMC-2000), from Ametek (Oak Ridge, TN). The UV-Vis Spectrophotometer (Cary 60) was purchased from Agilent (Santa Clara, CA). The experiments were carried out in a three-electrode cell configuration with a platinum working electrode and a platinum counter electrode. The potentials were measured versus Ag/AgCl in water. The pH was measured using a pH probe (Orion PerpHecT ROSS), which was calibrated prior to the experiment.

1.2. Procedure for extraction of reduced neutral red (NRH2)

The 4 mL aqueous solution of NRH2 was extracted with ethyl acetate (20 mL X 3 times). The combined organic layers were washed with water (20 mL X 5 times) to remove nicotinamide. The combined organic layers were dried over anhydrous magnesium sulfate, filtered, and concentrated under reduced pressure to afford NRH2 as a brown solid.

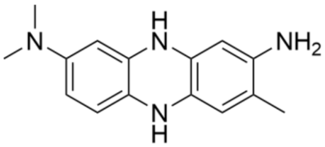

***N*^{2},*N*^{2},7-trimethyl-5,10-dihydrophenazine-2,8-diamine (NRH2)**

^{1}H NMR (400 MHz, DMSO) δ 7.81 (d, *J* = 9.5 Hz, 1H), 7.66 – 7.57 (m, 1H), 7.46 (dd, *J* = 9.5, 2.8 Hz, 1H), 6.89 (s, 1H), 6.81 (d, *J* = 2.7 Hz, 1H), 5.99 (s, 2H), 3.10 (s, 6H), 2.33 (s, 3H).
Minor peaks at δ 9.04, 8.69, 8.20, 8.16, 7.50 are from inseparable nicotinamide.

^{13}C NMR (101 MHz, DMSO) δ 150.95, 150.61, 145.52, 145.22, 136.96, 135.84, 131.01, 129.98, 129.03, 119.76, 102.78, 102.55, 40.06, 18.12.
Minor peaks at δ 166.89, 152.36, 149.14, 135.60, 130.13, 123.87 are from inseparable nicotinamide

HRMS (DART+) m/z Calc. 255.1610 $[C_{15}H_{19}N_4]^+$, Found. 255.1604

1.3. Procedure for control experiment

Procedure for electrochemical reduction of 1 M NA solution without NR in batch

Reactions were carried out with carbon felt (0.5 cm X 0.3 cm X 2 cm was immersed in the solution) cathode and a stainless steel wire anode in 5 mL H-cell with #9 O-ring equipped with anion exchange membrane. In the cathodic chamber, nicotinamide (488 mg, 4 mmol, 1 M) and lithium perchlorate (212 mg, 2 mmol, 0.5 M) were added into water (4.0 mL). In the anodic chamber equipped with a needle to prevent pressurization was placed lithium perchlorate solution (212 mg, 2 mmol, 0.5 M, 4.0 mL of water). The solution was bubbled with nitrogen for 10 min, after which the electrochemical potential was applied at room temperature by a constant current of –50 mA for 696 s (0.36 mmol of electrons).

2. Supplementary Notes

2.1. Setup of NR/NRH2 redox system for the electrochemical CO_2 capture and release in batch

2.1.1. General material information for electrochemical reaction setups in batch

1. 5 mL H-cell with #9 O-ring was purchased from Adams & Chittenden Scientific Glass Coop (Berkeley, CA).
2. Anion exchange membrane (Selemion AMV) was purchased from Asashi Glass (Japan).
3. Carbon felt (C100 AvCarb Soft Carbon Felt) was purchased from Fuel Cell Earth (Stoneham, MA).
4. DC supply (SPD1168X) was purchased from Siglent (Ohio).

2.1.2. Setup for the CO_2 release experiment in batch

We have used the identical setup as described in our previous work (see Figure S1 in supporting information).[1] An H-cell containing NRH2 solution that was saturated by CO_2 with C felt anode in the anodic chamber was connected to a DC power supply. The exiting gas stream from the anodic chamber was measured by a flow meter and FT-IR CO_2 sensor.

2.1.3. Setup for the CO_2 absorption experiments in batch

We have used the identical setup as described in our previous work (see Figure S2 in supporting information).[1] Gas cylinder (1, 4, and 15% CO_2 balanced by nitrogen, Airgas®) was connected to mass flow controller and the gas stream was introduced to an 8 mL reaction vial containing an NRH2 solution. The exiting gas stream after mixing with the solution was measured by a flow meter and FT-IR CO_2 sensor.

2.2. Setup of NR/NRH2 redox system for the electrochemical CO_2 capture and release in continuous flow

2.2.1. General material information for electrochemical reaction setups in continuous flow

1. 20 mL H-cell with #20 O-ring was purchased from Adams & Chittenden Scientific Glass Coop (Berkeley, CA).
2. Anion exchange membrane (Selemion AMV) was purchased from Asashi Glass (Japan).
3. Carbon felt (C100 AvCarb Soft Carbon Felt) was purchased from Fuel Cell Earth (Stoneham, MA).
4. DC supply (SPD1168X) was purchased from Siglent (OH).
5. Clear Scratch- and UV-Resistant Cast Acrylic Sheet (7/16) was purchased from McMaster-Carr (Aurora, OH).
6. Titanium foil, 0.025mm (0.001in) thick, 99.94% (metals basis) was purchased from Thermo Scientific (Waltham, MA).
7. Silicone rubber sheet 0.125in thick was purchased from Rogers corporation (Chandler, AZ).
8. Peristaltic tubing (Masterflex Versilon Chemical (06475)–14) was purchased from Masterflex (Radnor, PA).
9. Tygon tubing (ID 1/16in, OD 3/16in) was purchased from McMaster-Carr (Aurora, OH).
10. The peristaltic pump (Masterflex L/S) was purchased from Masterflex (Radnor, PA).
11. The syringe pump (Standard Infuse/Withdraw PHD ULTRA Syringe Pump) was purchased from Harvard Apparatus (Holliston, MA).

2.2.2. Setup for the electrochemical CO_2 capture and release in continuous flow

As shown in Supplementary Figure 6, a flow cell with C felt anode and cathode with an anion exchange membrane was connected to a DC power supply. The flow cell was connected to Tygon□ tubing (ID 1/16in, OD 3/16in) at a liquid inlet (bottom) and outlet (top) of anodic and cathodic chambers (thru-hole 1/16in). A peristaltic pump was used to deliver the electrolyte solution from the catholyte and anolyte reservoirs. The distal end of the tubing system from the cathodic chamber was connected to the anolyte reservoir and the tubing from the anodic chamber to the catholyte reservoir. The 15% CO_2 gas cylinder was connected to tubing and its stream was metered by a mass flow controller (MFC). The 15% CO_2 stream was introduced to the catholyte solution. At the distal end of the outlet tubing from the headspace of the anolyte reservoir, an FT-IR CO_2 sensor and a flow meter were installed to monitor the output gas during continuous flow operation.

The above setup was used to direct air capture with the addition of a syringe pump to compensate for water evaporation due to vigorous bubbling of air into the catholyte solution and a pH probe to monitor the pH change in catholyte solution during the operation.

3. Supplementary Discussion

3.1. Estimation of the hydroxide anion equivalent

$$\mathbf{NR + 2e^- + 2H_2O \rightarrow NRH2 + 2OH^-} \quad (1)$$

$$\mathbf{NRH + 2e^- + H_2O \rightarrow NRH2 + OH^-} \quad (2)$$

$$\mathbf{pH = pKa + \log\frac{[NR]}{[NRH]}} \quad (3)$$

$$\boldsymbol{n = \frac{2[NR]+[NRH]}{[NR]+[NRH]} = \frac{2\cdot 10^{pH-pKa}+1}{10^{pH-pKa}+1}} \quad (4)$$

pKa = 6.8 for the equilibrium between NR and NRH

Anolyte tank pH range = 6.2 – 7.1

n = 1.20 – 1.67

The two-electron reduction of the base form, NR, produces two equivalent of hydroxide anion (eq. 1) and the two-electron reduction of the acid form, NRH, produces one equivalent of hydroxide anion (eq. 2). The ratio of NR and NRH can be obtained from the measured pH value using the Henderson-Hasselbalch equation (eq. 3). The equivalent number (n) of hydroxide anion to NR/NRH by the bulk electrochemical reduction can be calculated as in eq. 4. From the pH range of 6.2 – 7.1 of the anolyte thank, hydroxide anion would be 1.67 – 1.20 equivalents to NR/NRH based on the experimentally measured pH value of the oxidized solution.

3.2. Energy calculation

$E_{cell} = -E_p^{red}$ (under N_2)+ E_p^{oxi} = 0.42 V (5)

1 eV = 96.49 kJ/mol, this corresponds to the Faraday constant (F = 96485 C/mol), where the energy in joules of n moles of particles each with energy E eV is equal to $E\cdot F\cdot n$.

The minimum energy requirement using 15% CO_2 in batch is:

$E = E_{cell} \times 96.49$ kJ/mol·eV × $(\varepsilon_{CO2})^{-1}$ = 81 kJ_e/mole of CO_2 (6)

Where the average value of ε_{CO2} for electron utilization is 0.50 in batch obtained in Figure 3b.

The minimum energy requirement using ambient air in batch is:

$E = E_{cell} \times 96.49$ kJ/mol·eV × $(\varepsilon_{CO2})^{-1}$ = 123 kJ_e/mole of CO_2 (7)

Where the average value of ε_{CO2} for electron utilization is 0.33 in batch obtained in Figure 5a.

$E_{cell} = -E_p^{red}$ (under CO_2)+ E_p^{oxi} = 0.26 V (8)

The minimum energy requirement using 15% CO_2 in continuous flow is:

$E = E_{cell} \times 96.49$ kJ/mol·eV × $(\varepsilon_{CO2})^{-1}$ = 35 kJ_e/mole of CO_2 (9)

Where the highest value of ε_{CO2} for electron utilization is 0.71 in continuous flow obtained in Figure 6c.

The minimum energy requirement using 15% CO_2 in continuous flow is:

$$E = E_{cell} \times 96.49\ \text{kJ/mol·eV} \times (\varepsilon_{CO2})^{-1} = 65\ \text{kJ}_e\text{/mole of } CO_2 \quad (10)$$

Where the highest value of ε_{CO2} for electron utilization is 0.386 in continuous flow obtained in Figure 6e.

3.3. Evaluation of other dye molecules as redox-active compounds

3.3.1. Toluidine blue

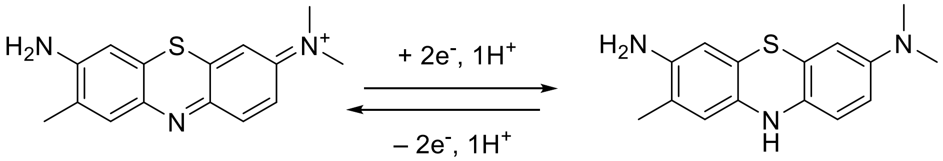

Electrochemical reduction

E_p^{red} = –0.233 V under N_2 vs Ag/AgCl

E_p^{red} = –0.202 V under CO_2 vs Ag/AgCl

Electrochemical oxidation

E_p^{oxi} = –0.144 V under CO_2 vs Ag/AgCl

$E_{cell} = -E_p^{red}$(under N_2) + E_p^{oxi} (under CO_2)= 0.089 V

Rapid reoxidation of the reduced form of toluidine blue when contacting air was observed.

3.3.2. Thionin

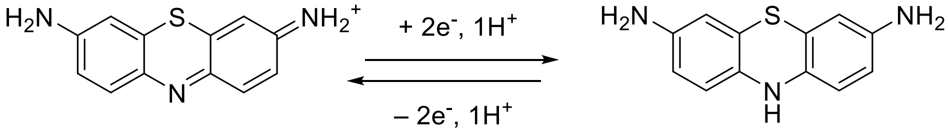

Electrochemical reduction

E_p^{red} = –0.233 V under N_2 vs Ag/AgCl

E_p^{red} = –0.240 and –0.304 V under CO_2 vs Ag/AgCl

Electrochemical oxidation

E_p^{oxi} = –0.072 V under CO_2 vs Ag/AgCl

$E_{cell} = -E_p^{red}$(under N_2) + E_p^{oxi} (under CO_2)= 0.232 V

Rapid reoxidation of the reduced form of thionin when contacting air was observed.

3.3.3. Safranin O

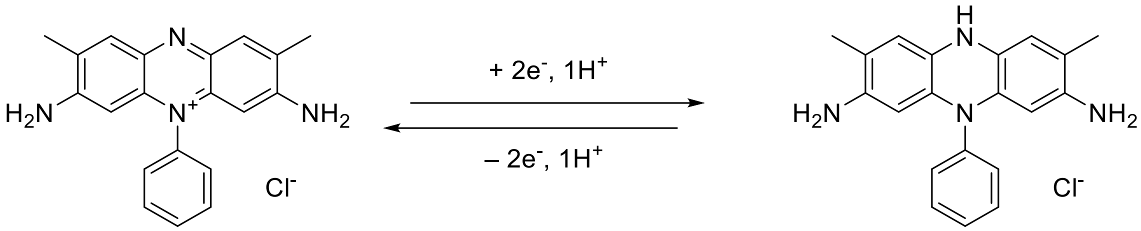

Electrochemical reduction

E_p^{red} = –0.443 and –0.552 V under N_2 vs Ag/AgCl

E_p^{red} = –0.369 and –0.485 V under CO_2 vs Ag/AgCl

Electrochemical oxidation

E_p^{oxi} = –0.424 and –0.303 V under CO_2 vs Ag/AgCl

$E_{cell} = -E_p^{red}$(under N_2) + E_p^{oxi} (under CO_2)= 0.249 V

Low solubility (< 5 mM) was observed in the presence of 1 M NA as a hydrotropic agent.

4. Supplementary Figures

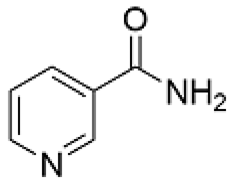

Nicotinamide (NA)

^{1}H NMR (400 MHz, H_2O+D_2O_salt) δ 8.61 (dd, *J* = 2.3, 0.9 Hz, 1H), 8.43 (dd, *J* = 5.1, 1.7 Hz, 1H), 7.97 – 7.89 (m, 1H), 7.31 (ddd, *J* = 8.1, 5.1, 0.9 Hz, 1H), 7.22 (s, 1H).

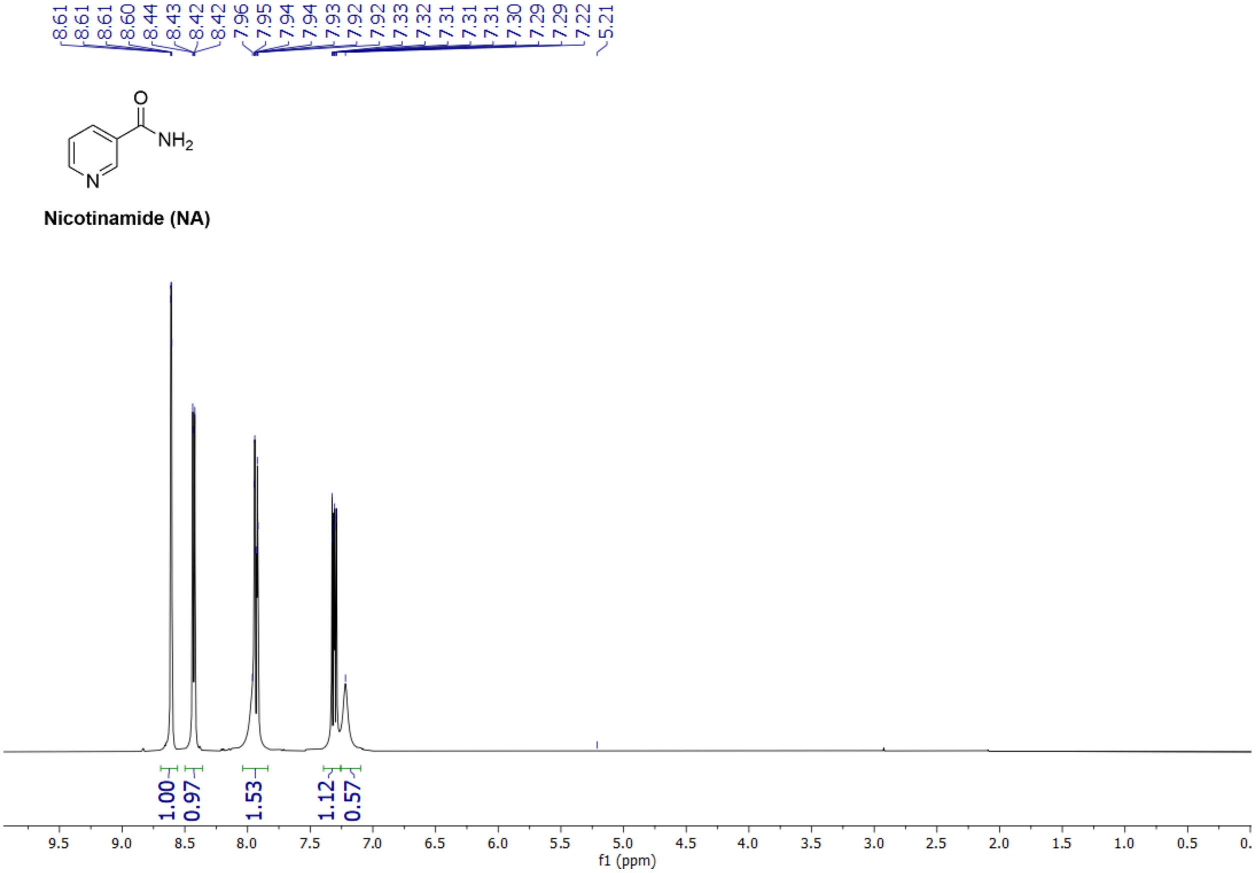

Supplementary Figure 1. H-NMR spectrum of nicotinamide in H_2O+D_2O

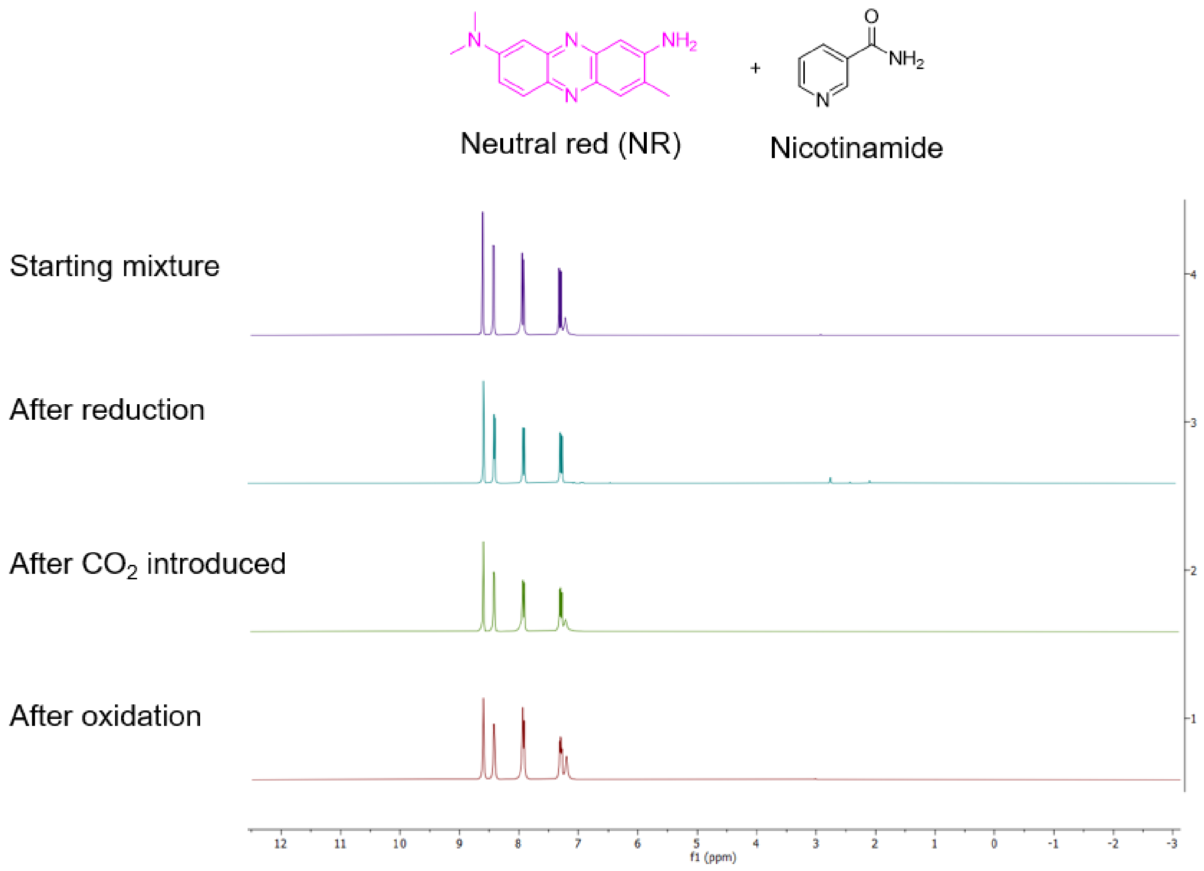

Supplementary Figure 2. ^{1}H-NMR spectra of the electrolyte solution of the starting material mixture, after electrochemical reduction, after CO_2 introduced to the solution, and after electrochemical oxidation.

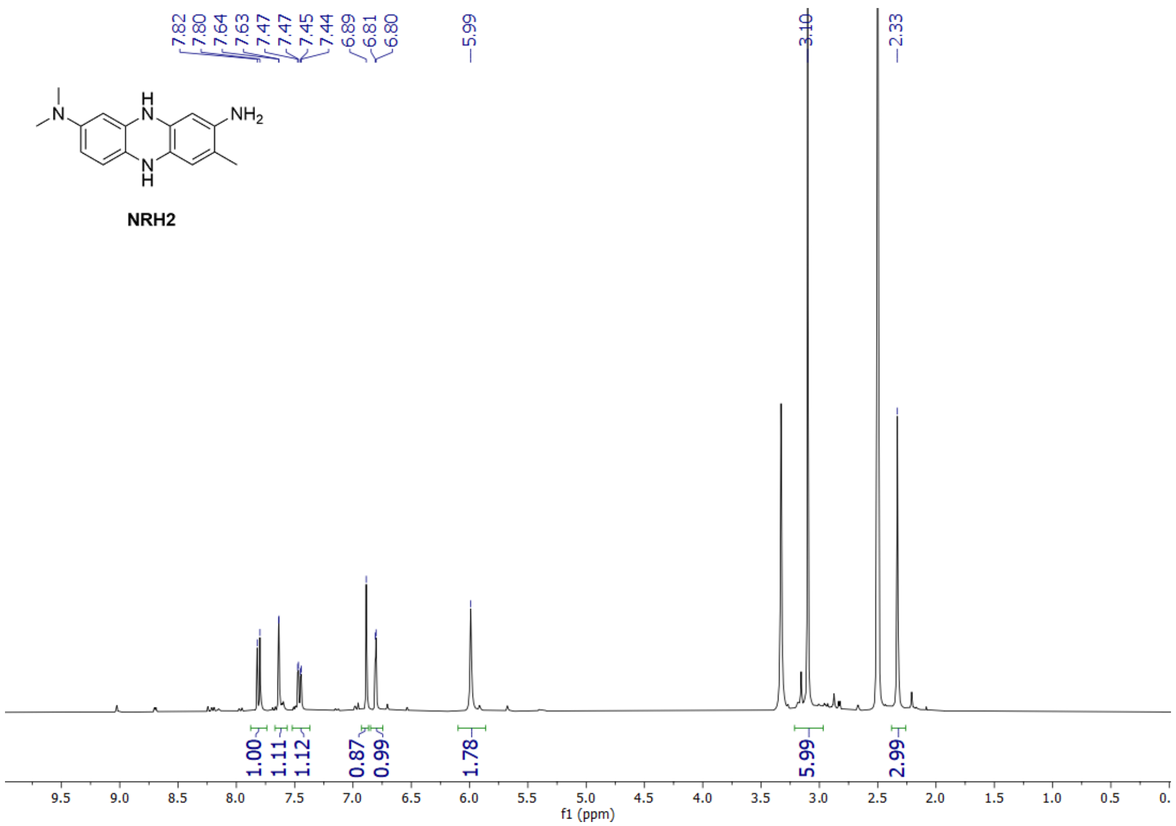

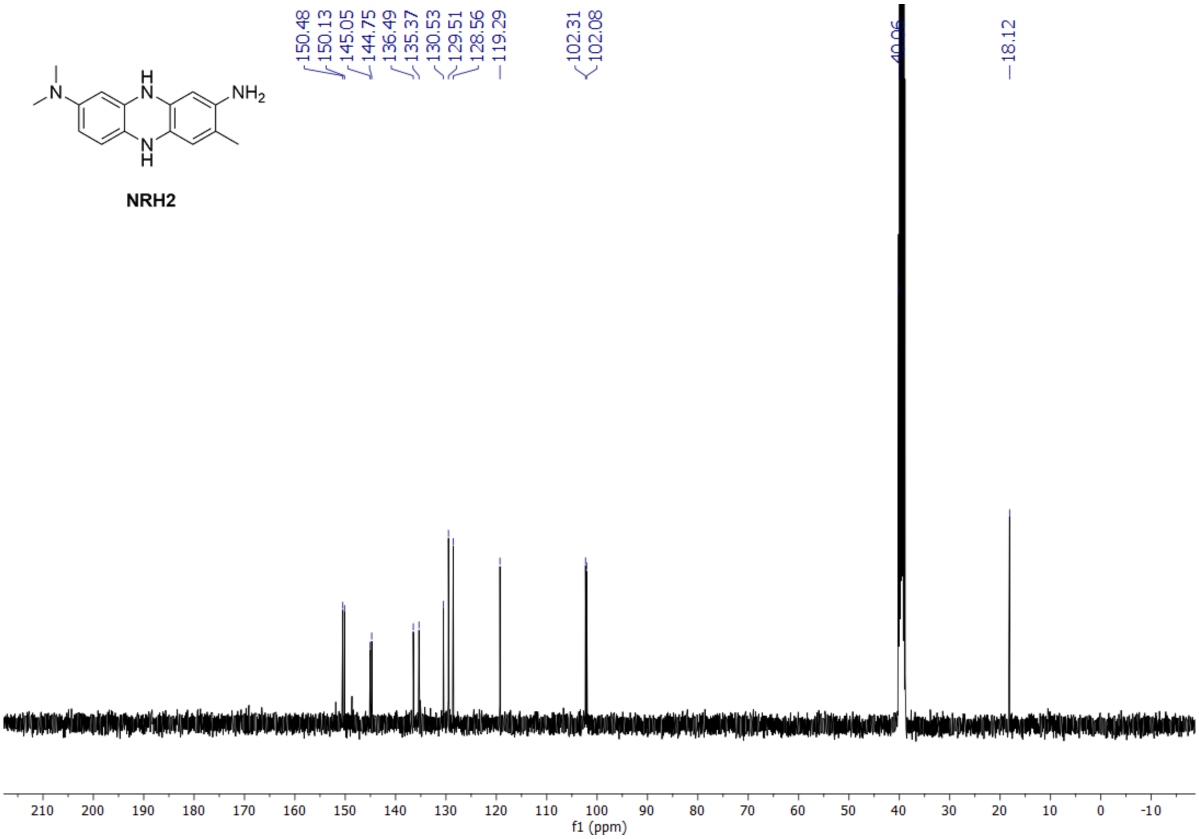

Supplementary Figure 3. ^{1}H- and ^{13}C-NMR spectra of NRH2 in DMSO

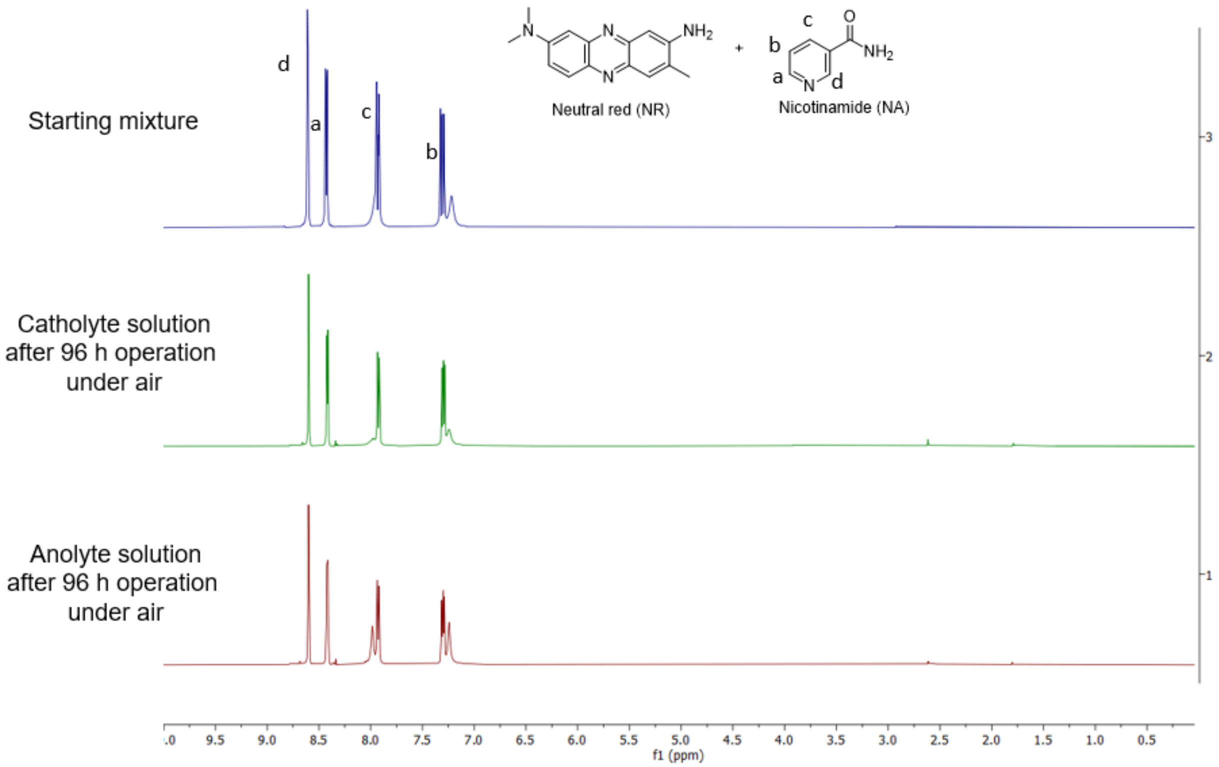

Supplementary Figure 4. ^{1}H NMR spectra were collected after 96 hours of operation under air confirming redox resistance of nicotinamide under the current conditions.

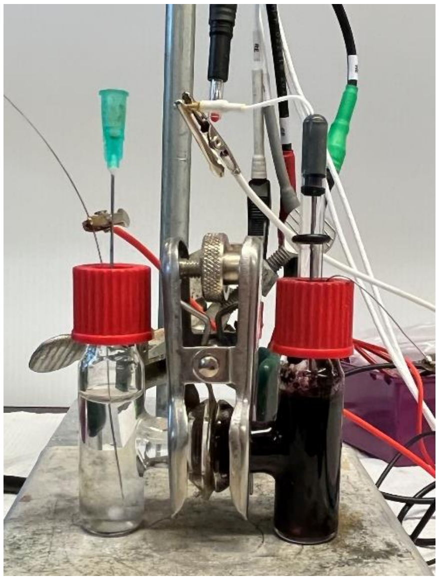

Supplementary Figure 5. Picture of H-cell

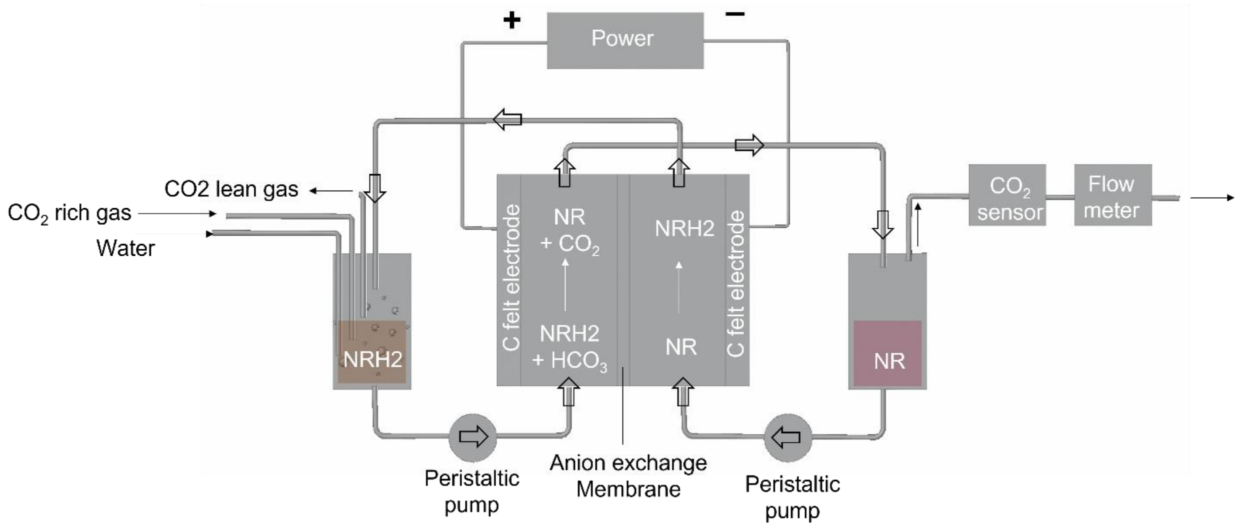

Supplementary Figure 6. Scheme of continuous flow electrochemical cell using NR/NRH2 redox cycle for CO_2 capture and release experiments.

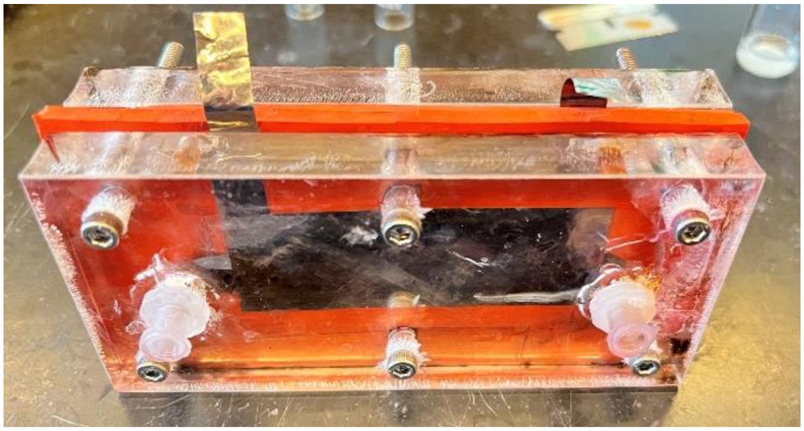

Supplementary Figure 7. Photograph of the assembled flow cell.

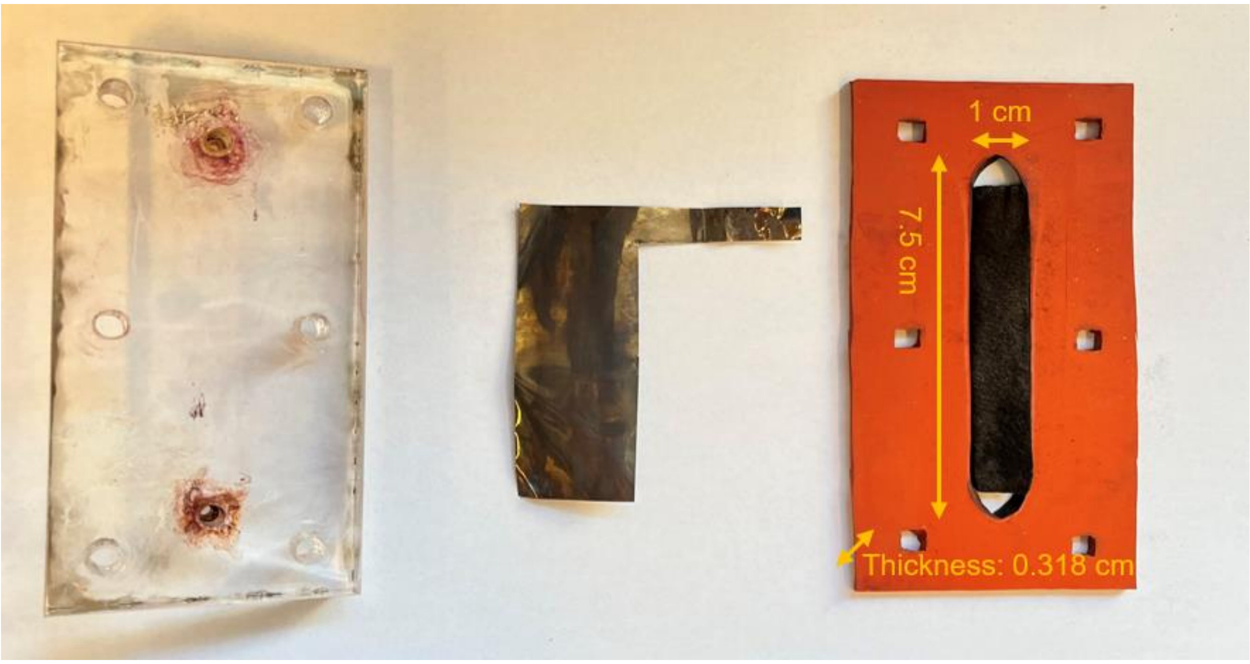

Supplementary Figure 8. Photograph of the flow cell.

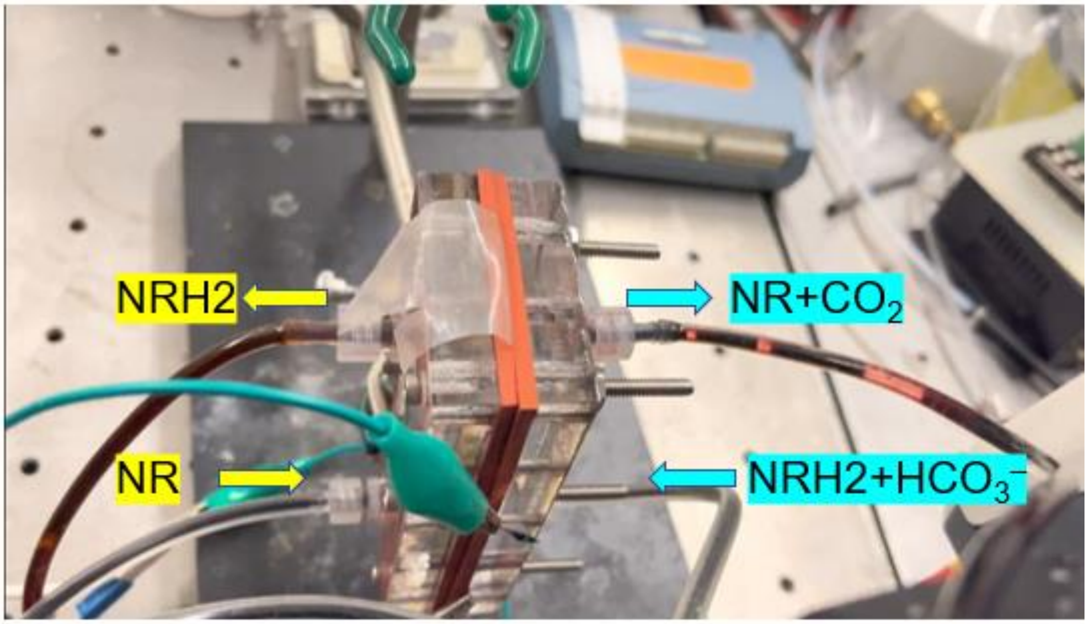

Supplementary Figure 9. Photograph of the flow cell during operation.

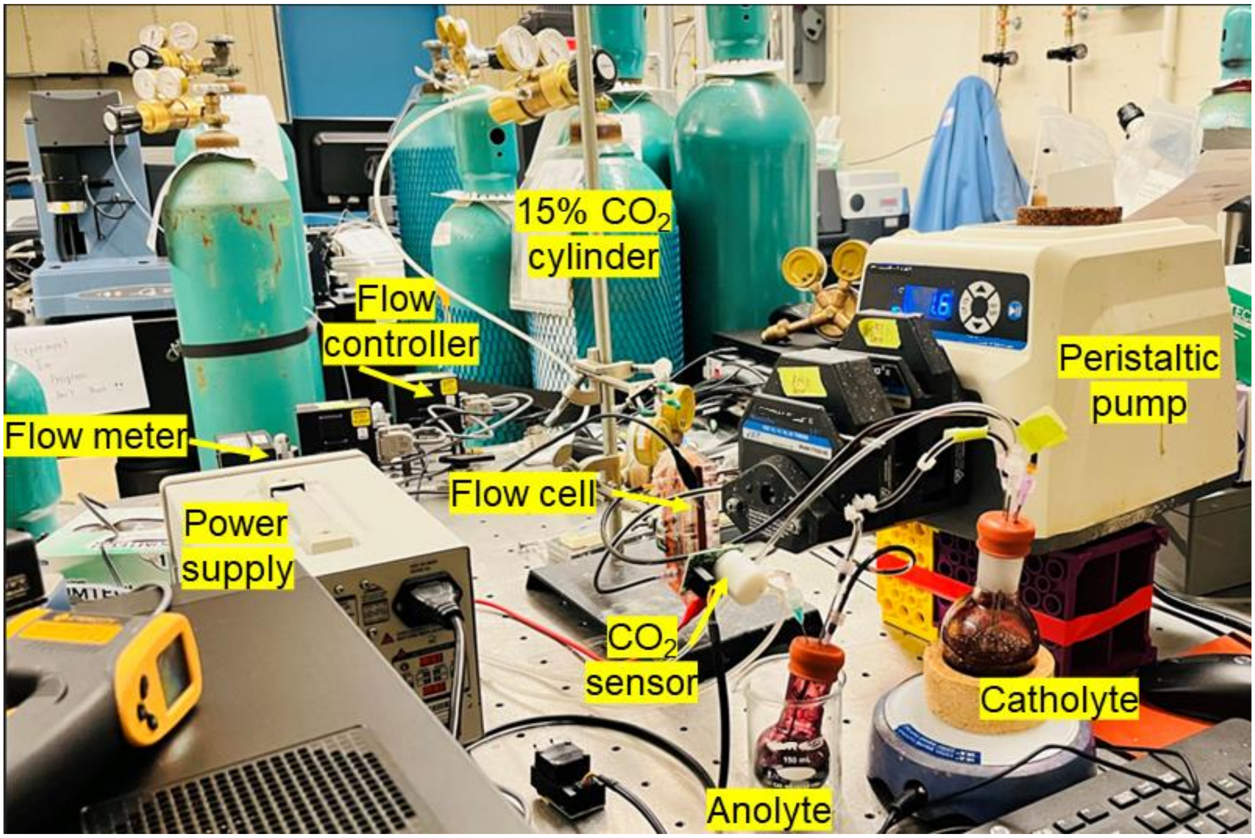

Supplementary Figure 10. Photograph of setup for electrochemical capture and release of CO_2 in continuous flow using 15% CO_2.

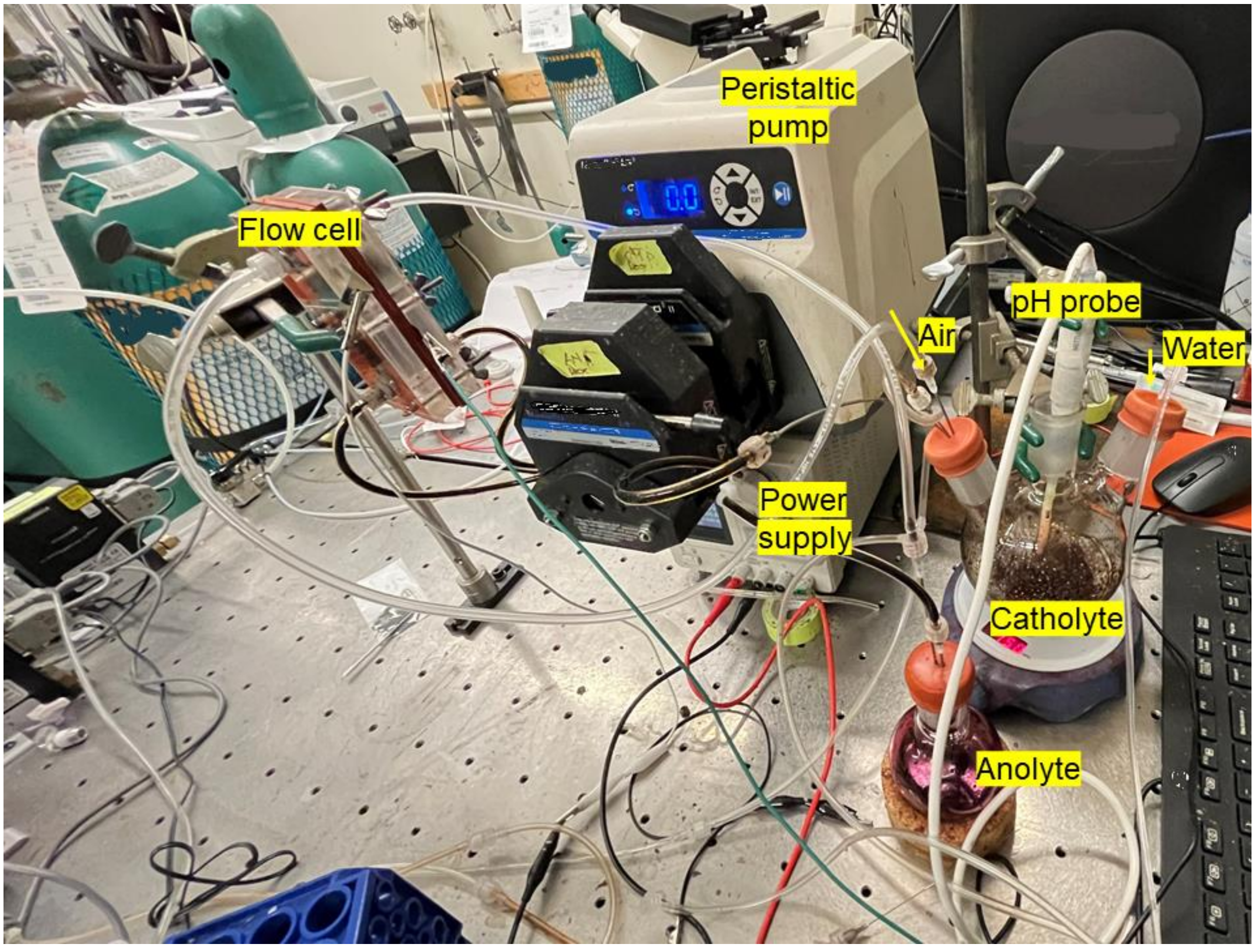

Supplementary Figure 11. Photograph of setup for electrochemical capture and release of CO_2 in continuous flow from the air.

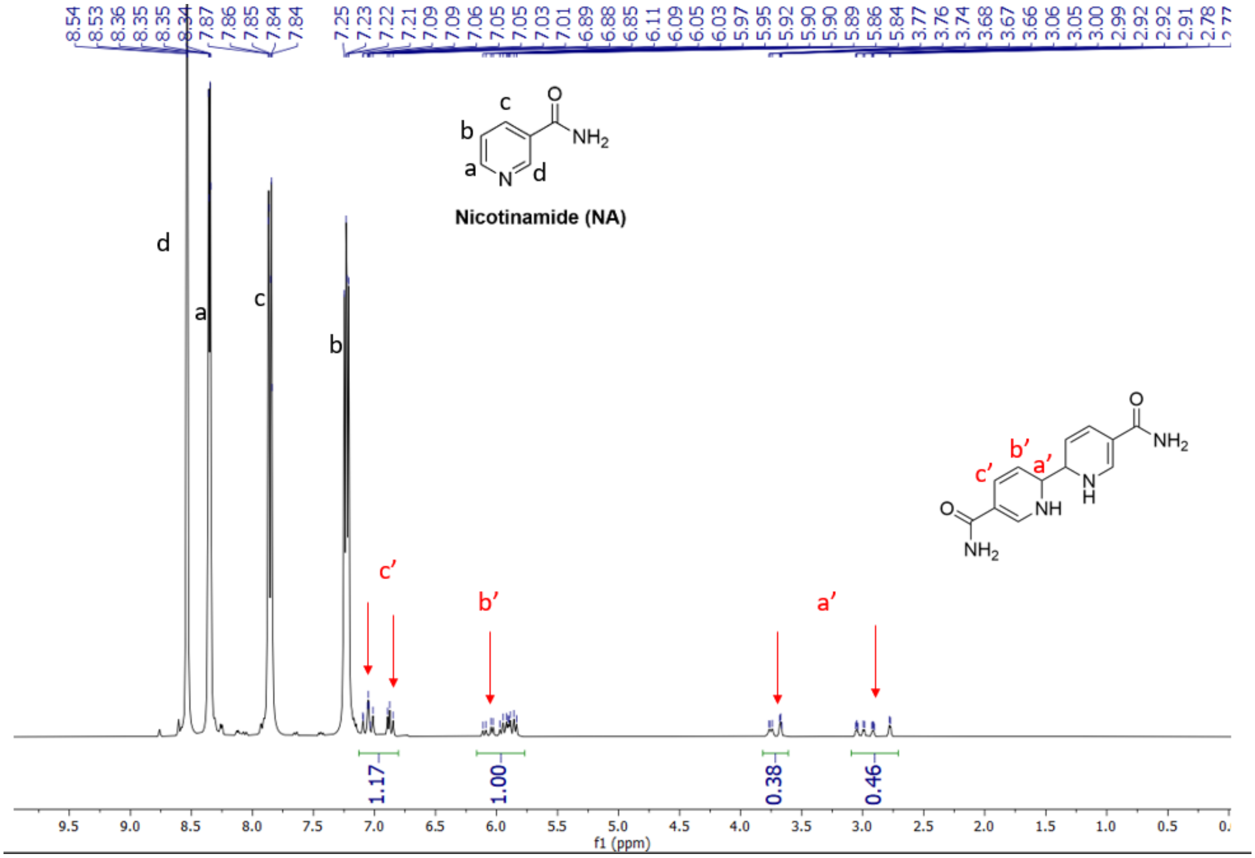

Supplementary Figure 12. ^{1}H-NMR spectra of the solution after reduction of the solution containing 1 M NA and 0.5 M $LiClO_4$.

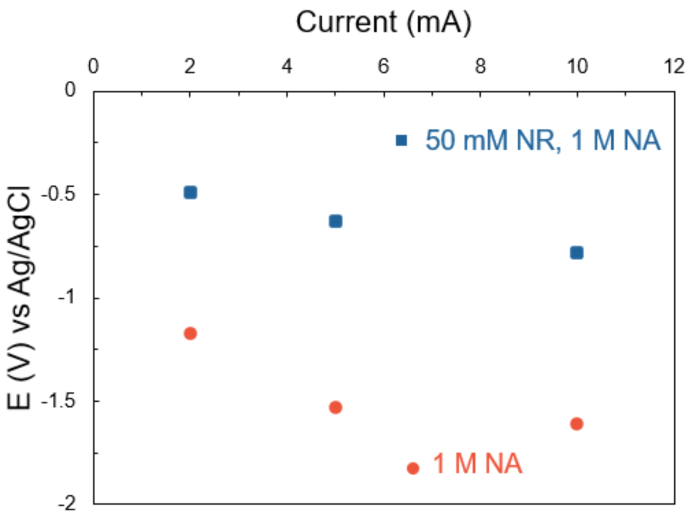

Supplementary Figure 13. Applied potentials vs Ag/AgCl of the solution containing 50 mM NR, 1 M NA, and 1 M KCl in water (blue squares), and 1 M NA and 1 M KCl in water (red dots) at constant currents of 2 mA, 5 mA, and 10 mA. The potential was recorded after 180 seconds of operation. Carbon felt (0.5 cm X 0.3 cm X 2 cm immersed in the solution) was used as a working electrode.

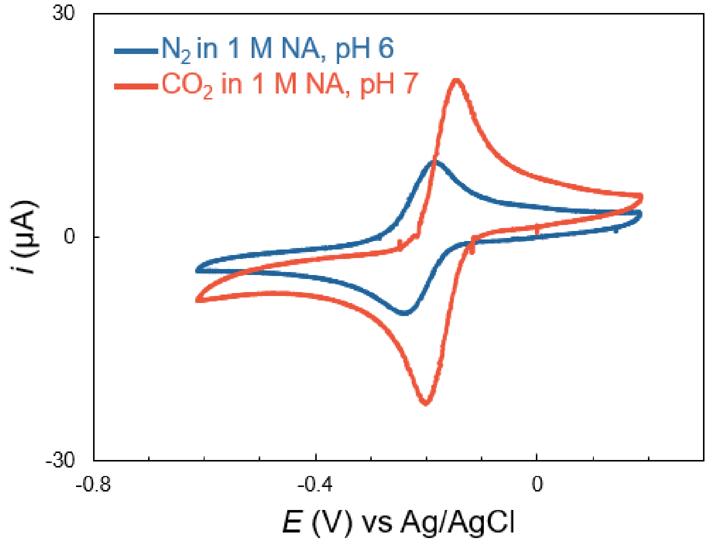

Supplementary Figure 14. The cyclic voltammograms of 10 mM of toluidine blue under nitrogen (blue curve) and CO_2 (red curve) in water with 0.1 M lithium perchlorate as a supporting electrolyte and 1 M nicotinamide as a hydrotropic agent. All CV curves were recorded at room temperature, with a glassy carbon working electrode, at a scan rate of 50 mV/s. Potentials were recorded versus Ag/AgCl as a reference electrode.

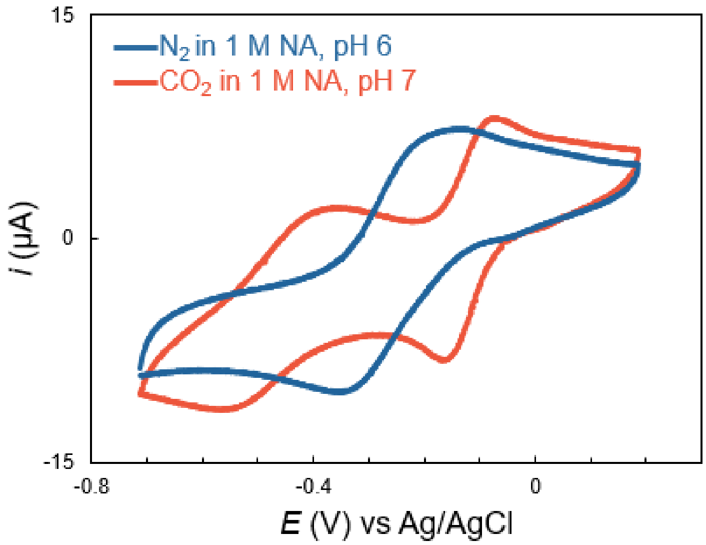

Supplementary Figure 15. The cyclic voltammograms of 10 mM of thionin under nitrogen (blue curve) and CO_2 (red curve) in water with 0.1 M lithium perchlorate as a supporting electrolyte and 1 M nicotinamide as a hydrotropic agent. All CV curves were recorded at room temperature, with a glassy carbon working electrode, at a scan rate of 50 mV/s. Potentials were recorded versus Ag/AgCl as a reference electrode.

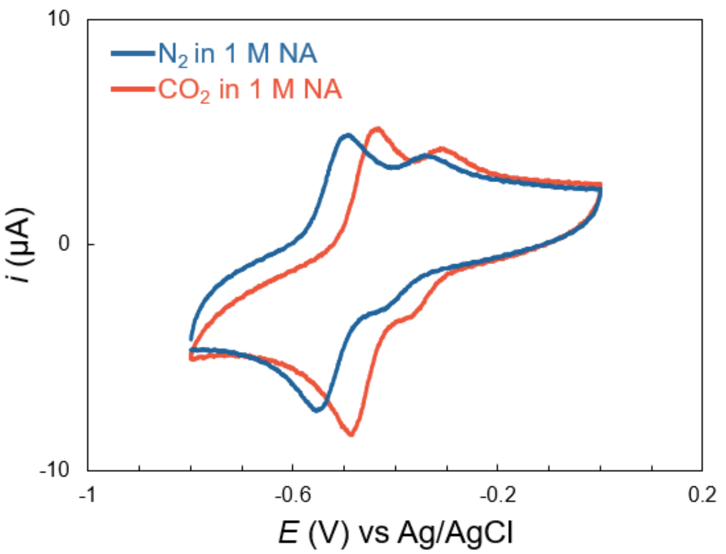

Supplementary Figure 16. The cyclic voltammograms of 5 mM of Safranin O under nitrogen (blue curve) and CO_2 (red curve) in water with 0.1 M lithium perchlorate as a supporting electrolyte and 1 M nicotinamide as a hydrotropic agent. All CV curves were recorded at room temperature, with a glassy carbon working electrode, at a scan rate of 50 mV/s. Potentials were recorded versus Ag/AgCl as a reference electrode.

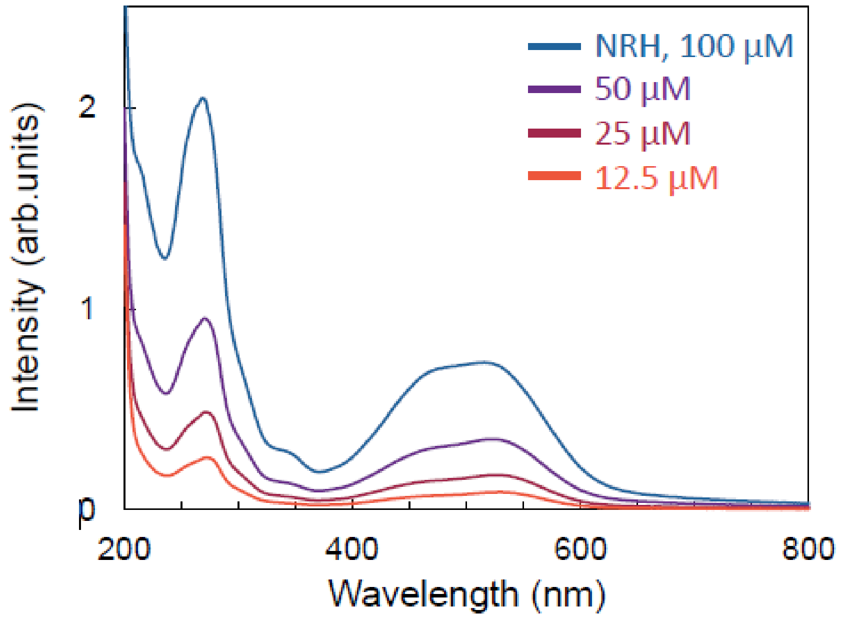

Supplementary Figure 17. UV-vis absorption spectroscopy of NRH aqueous solution in pH 6.6 phosphate buffer. Peaks at 525 nm are from the acid form, NRH, and peaks at 460 nm from the base form, NR.

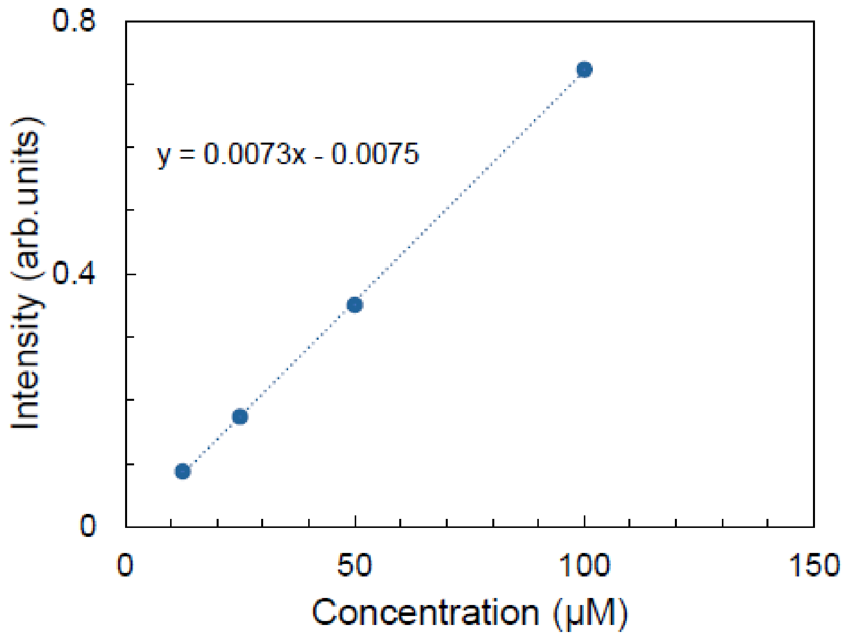

Supplementary Figure 18. Plot between NRH concentration and the UV-vis peak intensity at 525 nm.

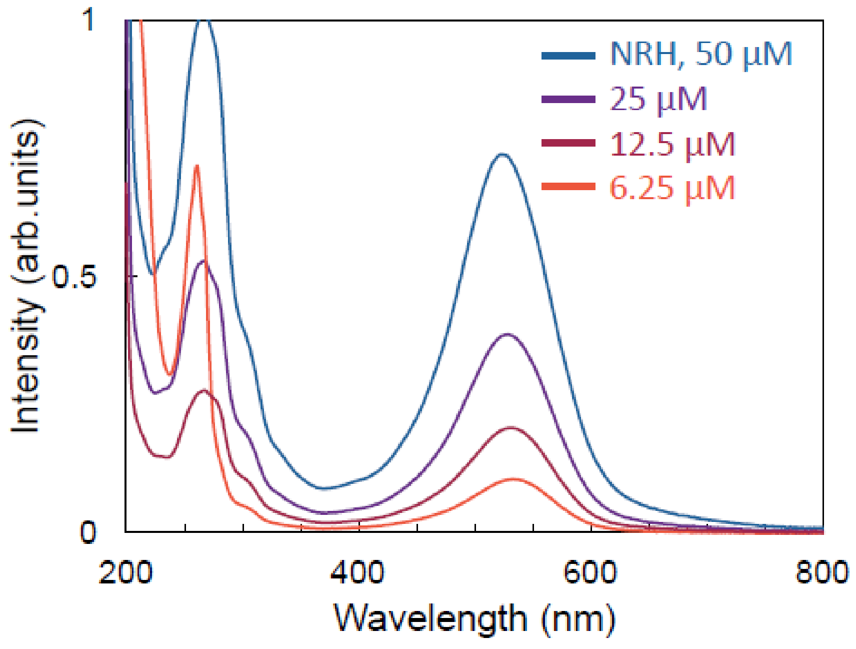

Supplementary Figure 19. UV-vis absorption spectroscopy of NRH aqueous solution in pH 1.3. Peaks at 525 nm show higher intensities than those measured in pH 6.6 (Supplementary Figure 17) with no peak at 460 nm.

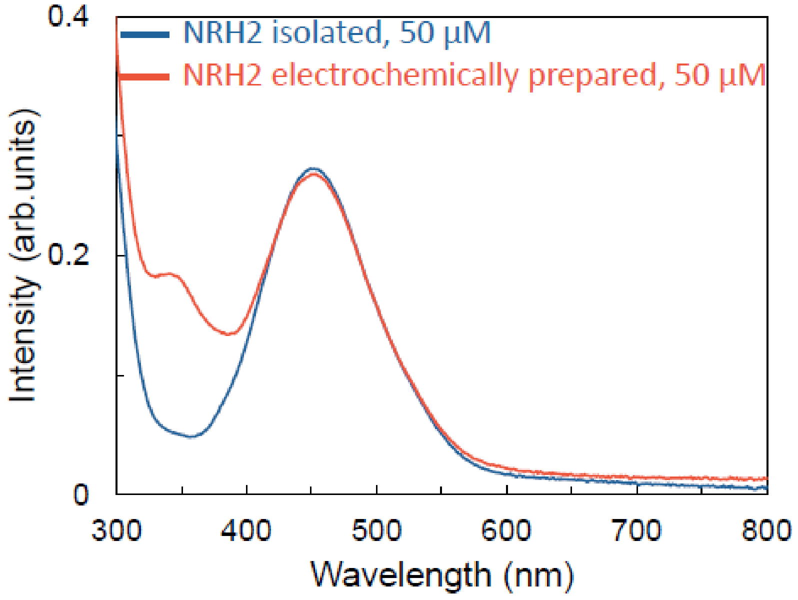

Supplementary Figure 20. UV-vis absorption spectroscopy of electrochemically prepared NRH2 solution and the solution prepared using the isolated NRH2.

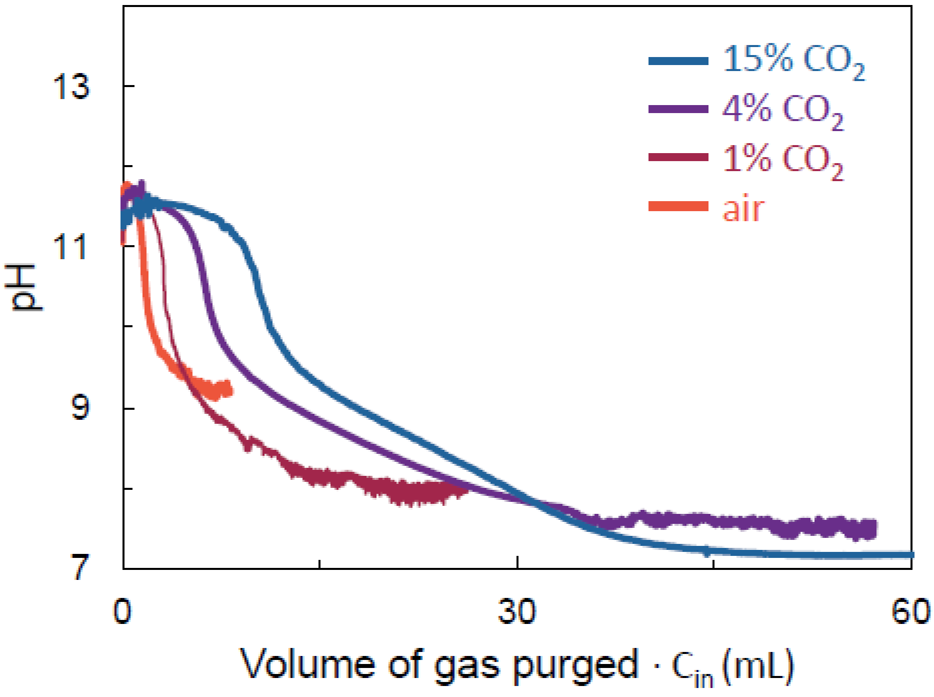

Supplementary Figure 21. Normalized pH changes while blowing gas streams of 1, 4, 15% CO_2, and air.

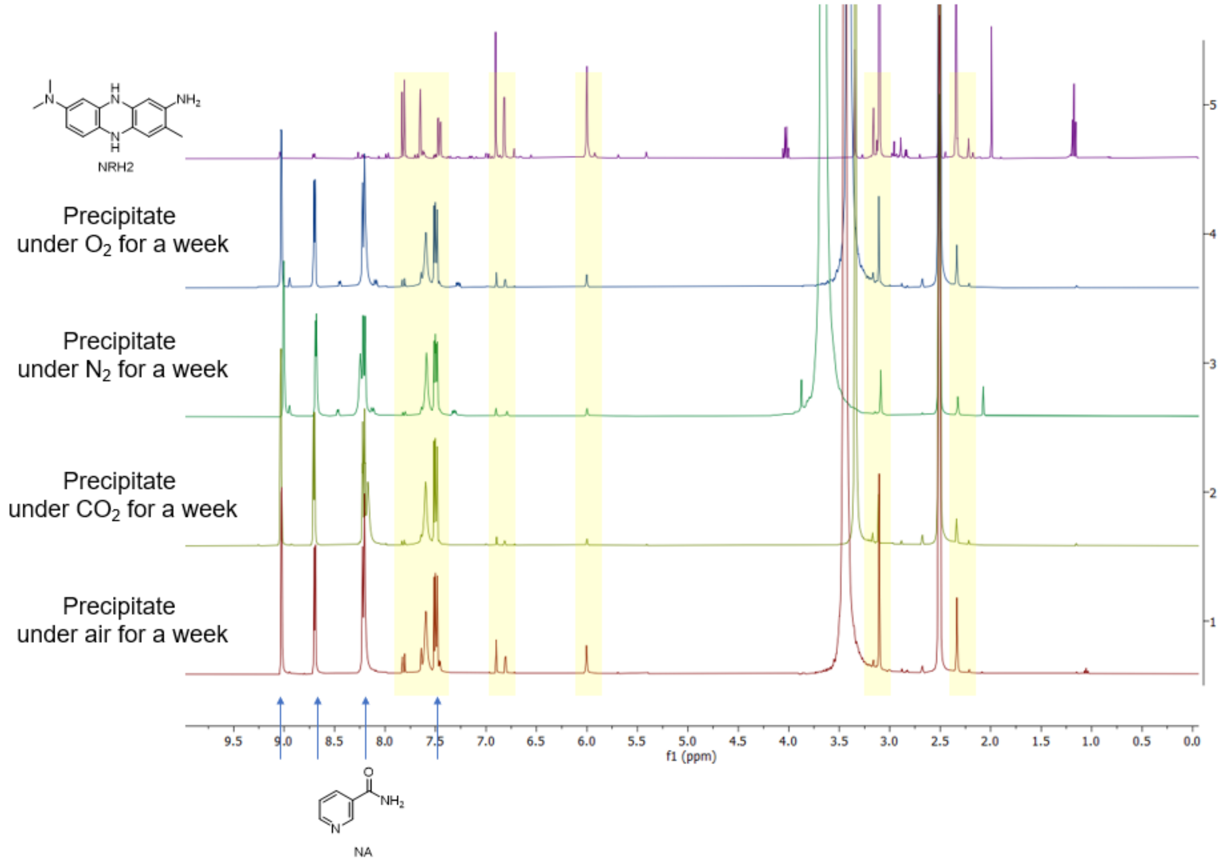

Supplementary Figure 22. ^{1}H-NMR spectrum of precipitate after stability tests under O_2, N_2, CO_2, and air for a week. Minor peaks from byproduct were observed at δ 8.94, 8.44, 8.09, 7.28 under O_2 and N_2. Procedure for stability test using UV-vis absorption spectroscopy were followed to prepare NMR samples. The solutions after stability tests were filtered and the precipitates were analyzed by ^{1}H-NMR.

Supplementary Table 1. Summary of electrochemical carbon capture methods for direct air capture

Entry	Redox-active compound	Description	Min. work of separation (kJ_e/mol)	Remarks
1	Neutral red (This work)	pH swing in aqueous solution	Est. 35 (15% CO_2) Est. 64 (air)	O_2 insensitive Direct air capture demonstrated
2	DSPZ[2]	pH swing in aqueous solution	1.9 (46.5% CO_2) Est. 30-75 (air)	O_2 sensitivity limits DAC applicability
3	DHPS[3]	pH swing in aqueous solution	Est. 21.6 (15% CO_2)	O_2 sensitivity limits DAC applicability
4	1-AP nitrate[1]	pH swing in aqueous solution	Est. 162 (air)	
5	9,10-phenanthrenequinone[4]		Est. 34 (0.24 M CO_2 in ACN)	O_2 sensitivity limits DAC applicability; Requires organic solvent or ionic liquid
6	2,6-di-*tert*-butyl-1,4-benzoquinone[5]		Est. 61 (0.5% CO_2)	O_2 sensitivity limits DAC applicability
7	N-propyl-4,4′-bipyridinium[6]		Est. 19 (2.8 mM CO_2 in ACN)	ACN solvent
8	4,4′-bipyridine[7]		Est. 67 (CO_2 saturated solution in BMP-TFSI)	1,2-dichloroethane solvent
9	EMAR[8]	CO_2 sorbent: Ethylene diamine; Electrochemical swing between Cu(II) and Cu(0)	16 (15% CO_2)	Repetitive polarity switches; Aqueous solution
10	Co(III) complex[9]	CO_2 sorbent: cyclopentadienyl indenyl Co(III) complex	Est. 34	CO_2 reduction; Irreversible decomposition
11	benzyldisulfide[10]	CO_2 sorbent: benzylthiolate	Est. 193 (5 mM CO_2 In BMP-TFSI)	Demonstrated in DMF
12	Cu(II) complex[11]	CO_2 sorbent: 1,2-dimethylimidazole; Electrochemical swing between Cu(II) and Cu(I)	Est. 27-65 (15% CO_2)	Low cyclic stability due to unstable Cu(I) complexes
13	Polyanthraquinone[12]		113 (400 ppm CO_2)	O_2 sensitivity limits current DAC applicability
14	PCET: MnO_2[13]	pH swing K_2CO_3 capture solution	33 (15% CO_2)	Same benefits and downsides as traditional K_2CO_3

DSPZ: sodium 3,30-(phenazine-2,3-diylbis(oxy))bis(propane-1-sulfonate)

DHPS: 7,8-dihydroxyphenazine-2-sulfonic acid

ACN: acetonitrile

BMP-TFSI: 1-Butyl-1-methylpyrrolidinium bis(trifluoromethylsulfonyl)imide

6. Supplementary References

1. Seo, H., Rahimi, M. & Hatton, T. A. Electrochemical Carbon Dioxide Capture and Release with a Redox-Active Amine. *J. Am. Chem. Soc.* **144**, 2164–2170 (2022).
2. Jin, S., Wu, M., Gordon, R. G., Aziz, M. J. & Kwabi, D. G. pH swing cycle for CO_2 capture electrochemically driven through proton-coupled electron transfer. *Energy Environ. Sci.* **13**, 3706–3722 (2020).
3. Xie, H. *et al.* Low-energy-consumption electrochemical CO_2 capture driven by biomimetic phenazine derivatives redox medium. *Appl. Energy* **259**, 114119 (2020).
4. Mizen, M. B. & Wrighton, M. S. Reductive Addition of CO_2 to 9,10-Phenanthrenequinone. *J. Electrochem. Soc.* **136**, 941–946 (1989).
5. Scovazzo, P., Poshusta, J., DuBois, D., Koval, C. & Noble, R. Electrochemical Separation and Concentration of <1% Carbon Dioxide from Nitrogen. *J. Electrochem. Soc.* **150**, D91 (2003).
6. Ishida, H., Ohba, T., Yamaguchi, T. & Ohkubo, K. Interaction between CO_2 and Electrochemically Reduced Species of *N*-propyl-4,4′-bipyridinium Cation. *Chem. Lett.* **23**, 905–908 (1994).
7. Ranjan, R. *et al.* Reversible Electrochemical Trapping of Carbon Dioxide Using 4,4′-Bipyridine That Does Not Require Thermal Activation. *J. Phys. Chem. Lett.* **6**, 4943–4946 (2015).
8. Stern, M. C., Simeon, F., Herzog, H. & Hatton, T. A. Post-combustion carbon dioxide capture using electrochemically mediated amine regeneration. *Energy Environ. Sci.* **6**, 2505 (2013).
9. DuBOIS, D. L., Miedaner, A., Bell, W. & Smart, J. C. ELECTROCHEMICAL CONCENTRATION OF CARBON DIOXIDE. in *Electrochemical and Electrocatalytic Reactions of Carbon Dioxide* 94–117 (Elsevier, 1993). doi:10.1016/B978-0-444-88316-2.50008-5.
10. Singh, P. *et al.* Electrochemical Capture and Release of Carbon Dioxide Using a Disulfide–Thiocarbonate Redox Cycle. *J. Am. Chem. Soc.* **139**, 1033–1036 (2017).
11. Boualavong, J. & Gorski, C. A. Electrochemically Mediated CO_2 Capture Using Aqueous Cu(II)/Cu(I) Imidazole Complexes. *ACS EST Eng.* **1**, 1084–1093 (2021).

12. Hemmatifar, A., Kang, J. S., Ozbek, N., Tan, K. & Hatton, T. A. Electrochemically Mediated Direct CO_2 Capture by a Stackable Bipolar Cell. *ChemSusChem* (2022) doi:10.1002/cssc.202102533.
13. Rahimi, M., Catalini, G., Puccini, M. & Hatton, T. A. Bench-scale demonstration of CO_2 capture with an electrochemically driven proton concentration process. *RSC Adv.* **10**, 16832–16843 (2020).
